# MicroRNA-4735-3p Facilitates Ferroptosis in Clear Cell Renal Cell Carcinoma by Targeting SLC40A1

**DOI:** 10.1155/2022/4213401

**Published:** 2022-05-19

**Authors:** Cairong Zhu, Zhiquan Song, Zhen Chen, Tianqi Lin, Haili Lin, Zhenqiang Xu, Fen Ai, Shijiao Zheng

**Affiliations:** ^1^Hemodialysis Room, The 7th Hospital of Wuhan, Wuhan, 430071 Hubei, China; ^2^Department of Stomatology, First People's Hospital of Zaoyang City, Zaoyang, 441100 Hubei, China; ^3^Department of Emergency, The Central Hospital of Wuhan, Tongji Medical College, Huazhong University of Science and Technology, Wuhan, 430014 Hubei, China; ^4^Department of Urology, Zhangzhou Affiliated Hospital of Fujian Medical University, Zhangzhou, 363000 Fujian, China

## Abstract

**Objective:**

Clear cell renal cell carcinoma (ccRCC) is the major histopathological subtype of renal cancer, and ferroptosis is implicated in the pathogenesis of ccRCC. The present study was aimed at investigating the role and underlying mechanisms of microRNA-4735-3p (miR-4735-3p) in ccRCC.

**Methods:**

Human ccRCC cell lines were transfected with the miR-4735-3p mimic or inhibitor to manipulate the expression of miR-4735-3p. Cell proliferation, colony formation, cell migration, cell invasion, cell death, oxidative stress, lipid peroxidation, and iron metabolism were determined. To validate the necessity of solute carrier family 40 member 1 (SLC40A1), human ccRCC cell lines were overexpressed with SLC40A1 using adenoviral vectors.

**Results:**

miR-4735-3p expression was reduced in human ccRCC tissues and cell lines but elevated upon ferroptotic stimulation. The miR-4735-3p mimic increased, while the miR-4735-3p inhibitor decreased oxidative stress, lipid peroxidation, iron overload, and ferroptosis of human ccRCC cell lines. Mechanistic studies identified SLC40A1 as a direct target of miR-4735-3p, and SLC40A1 overexpression significantly attenuated iron overload and ferroptosis in the miR-4735-3p mimic-treated human ccRCC cell lines.

**Conclusion:**

miR-4735-3p facilitates ferroptosis and tumor suppression in ccRCC by targeting SLC40A1.

## 1. Introduction

Renal cell carcinoma (RCC) is the most frequent renal cancer derived from renal tubular epithelial cells, among which clear cell RCC (ccRCC) is the major histopathological subtype with higher invasive ability and relapse risk, accounting for approximately 80%-90% of all RCC cases. Radical surgery is the primary strategy to treat ccRCC; however, 20%-30% of patients present with metastases at initial diagnosis. And these patients acquire limited benefits from conventional radiotherapy, chemotherapy, and immunotherapy, instead suffering from severe side effects [1, 2]. Ferroptosis is a novel form of programmed cell death mainly caused by an iron-dependent accumulation of toxic lipid reactive oxygen species (ROS) and plays critical roles in tumor progression [3–6]. Recent studies have shown that ferroptosis is implicated in the pathogenesis of ccRCC and that targeting ferroptosis provides novel insights to treat human ccRCC [7–9]. Woo et al. demonstrated that inducing ferroptosis dramatically decreased the viability of human ccRCC cells [10]. Glutathione redox system helps to scavenge intracellular free radicals and suppresses ferroptosis [11]. Miess et al. revealed that inhibition of glutathione synthesis triggered a significantly increased death of ccRCC cells and blocked tumor growth [12]. In addition, the expressions of various ferroptosis-related genes can accurately predict the prognosis and survival outcome in ccRCC patients and are identified as potential prognostic biomarkers and therapeutic targets [11, 13]. Moreover, triggering ferroptosis also enhances the efficiency of chemotherapy and immunotherapy for some refractory cases [14, 15]. Based on these findings, it is promising and highly desirable to treat human ccRCC through inducing ferroptosis.

Intracellular iron homeostasis is essential for cell survival, while iron overload contributes to ROS overproduction through the Fenton reaction, thereby facilitating ferroptosis and tumor suppression [16–19]. Regulation of iron metabolism is primarily orchestrated by transferrin- (TF-)/transferrin receptor- (TFR-) mediated uptake of extracellular iron and solute carrier family 40 member 1- (SLC40A1, also known as FPN1) mediated iron export [20]. Of note, SLC40A1 is the only discovered iron export protein in mammals, and inhibiting SLC40A1 induces ferroptosis and blocks growth of multiple tumors. Tang et al. recently demonstrated that facilitating SLC40A1 degradation dramatically increased intracellular iron content and subsequently induced ferroptosis and tumor suppression in lung cancer [21]. MicroRNAs (miRs), a class of small noncoding RNAs, negatively regulate gene expressions through binding to the complementary sequences in the 3′-untranslated region (UTR) of targeted mRNAs and are implicated in the pathogenesis of ccRCC [22, 23]. Li et al. revealed that miR-153-5p expression was elevated and correlated to unfavorable clinical features in ccRCC and that miR-153-5p depletion significantly suppressed the proliferation and metastasis of ccRCC [24]. Besides, Kalantzakos et al. demonstrated that the viability, migration, and invasion of ccRCC cell lines were attenuated by miR-424-5p [25]. And our recent study also identified miR-99b and miR-99b∗ as tumor suppressors of human ccRCC cell lines [26]. miR-4735-3p plays important roles in diverse biological processes of different cancers, including prostate cancer, bladder cancer, ovarian cancer, and lung cancer [27–30]. The present study was aimed at investigating the role and underlying mechanisms of miR-4735-3p in ccRCC.

## 2. Materials and Methods

### 2.1. Reagents

Necrostain-1 (Nec-1, N9037), Z-VAD-FMK (Z-VAD, V116), chloroquine (CQ, C6628), 2′,7′-dichlorofluorescin diacetate (DCFH-DA, D6883), dihydroethidium (37291), and Iron Assay Kit (MAK025) were purchased from Sigma-Aldrich (St. Louis, MO, USA). Erastin (S7242), RSL3 (S8155), ferrostain-1 (Fer-1, S7243), liproxstatin-1 (Lip-1, S7699), deferoxamine mesylate (DFO, S5742), and ciclopirox ethanolamine (CPX, S3019) were obtained from Selleck Chemicals (Houston, TX, USA). Lactate Dehydrogenase (LDH) Assay Kit (ab102526) and Lipid Peroxidation Assay Kit (ab118970) were obtained from Abcam (Cambridge, UK). Lipid Peroxidation Assay Kit (A106) was purchased from Nanjing Jiancheng Bioengineering Institute (Nanjing, China). Hieff Trans™ Liposomal Transfection Reagent (40802ES02) was purchased from Yeasen Biotechnology Co., Ltd. (Shanghai, China). Colorimetric CytoSelect™ 24-Well Cell Migration and Invasion Assay Kit (CBA-100) was purchased from Cell Biolabs (San Diego, CA, USA). The miR-4735-3p mimic (miR10019861-1-5), mimic control (miR1N0000001-1-5), miR-4735-3p inhibitor, and inhibitor control (miR2N0000001-1-5) were obtained from RiboBio Co., Ltd. (Guangzhou, China). Adenovirus carrying full-length human SLC40A1 or Ctrl plasmid were synthetized by Genechem Co., Ltd. (Shanghai, China). Anti-SLC40A1 (ab58695) and anti-glyceraldehyde-3-phosphate dehydrogenase (GAPDH, ab8245) were purchased from Abcam.

### 2.2. Cell Lines and Culture

Human ccRCC cell lines 796-O and A498 and human normal kidney cell line HK-2 were cultured in RPMI 1640 medium containing 10% fetal bovine serum as we previously described [26]. To stimulate ferroptosis in human ccRCC cell lines, 796-O or A498 cells were incubated with erastin (5 *μ*mol/L) or RSL3 (1 *μ*mol/L) for 24 h [9]. To overexpress miR-4735-3p, 796-O or A498 cells were transfected with the miR-4735-3p mimic (50 nmol/L) or mimic control for 6 h using Hieff Trans™ Liposomal Transfection Reagent and then were cultured with fresh medium for an additional 72 h [26]. To inhibit endogenous miR-4735-3p, the cells were transfected with the miR-4735-3p inhibitor (100 nmol/L) or inhibitor control [26]. To clarify the form of cell death in the miR-4735-3p mimic-treated ccRCC cell lines, these cells were incubated with Fer-1 (2 *μ*mol/L), Lip-1 (1 *μ*mol/L), DFO (100 *μ*mol/L), CPX (5 *μ*mol/L), Nec-1 (2 *μ*mol/L), Z-VAD (5 *μ*mol/L), or CQ (25 *μ*mol/L) for 24 h [7, 9, 21]. To verify the necessity of SLC40A1, 796-O or A498 cells were preinfected with adenovirus carrying full-length human SLC40A1 or Ctrl plasmid at a multiplicity of infection of 10 for 6 h and then incubated in fresh RPMI 1640 medium for 24 h before transfection. All cells were harvested at 72 h after transfection except specific annotation.

### 2.3. RNA Isolation and Quantitative Real-Time PCR

Total RNA was extracted and converted to cDNA according to standard protocols [31]. As we previously described, gene expression was calculated with U6 and GAPDH as the internal controls for miR-4735-3p and SLC40A1, using the 2^-*ΔΔ*Ct^ method [26].

### 2.4. Cell Viability and Colony Formation

After transfection with the miR-4735-3p mimic or inhibitor for 72 h, the viability of human ccRCC cell lines was determined using the MTT method [26, 32, 33]. In brief, the cells were incubated with MTT solution (0.5 mg/mL) at 37°C for 4 h, and then, the absorbance was measured at 490 nm. To evaluate colony formation, the cells were seeded in 6-well plates and cultured at 37°C for 14 days. Next, the cells were fixed with paraformaldehyde for 15 min, stained with 0.1% crystal violet for 30 min, and rinsed and counted in a blinded manner [34, 35].

### 2.5. Cell Death Analysis

LDH releases to the medium were measured to evaluate cell death as previously described [36]. Briefly, culture medium was centrifuged to remove cell debris and then incubated with LDH Reaction Mix (50 *μ*L). Next, the optical density was measured at 450 nm according to the manufacturer's instructions.

### 2.6. Cell Migration and Invasion Assay

Cell migration and invasion were evaluated using Transwell assay as described by us and other laboratories with a colorimetric CytoSelect™ 24-Well Cell Migration and Invasion Assay Kit [9, 37]. Briefly, 3 × 10^4^ 786-O cells and 1 × 10^5^ 498 cells were seeded in the upper chambers of the Transwell assay inserts with 200 *μ*L serum-free RPMI 1640 medium, and the lower chambers were added with 500 *μ*L RPMI 1640 medium containing 10% fetal bovine serum. After 12 h, nonmigratory cells in the interior of the inserts were removed by a cotton swab, and then, the inserts were incubated with 400 *μ*L cell stain solution for 10 min at room temperature. Next, the stained inserts were carefully washed, dried, and incubated with 200 *μ*L extraction solution for 10 min at room temperature, and then, the optical density was measured at 560 nm and calculated as the migrative cells. To evaluate cell invasion, the inserts coated with a dried basement membrane matrix in the upper surface was used, and the other assays were performed as the same steps mentioned above.

### 2.7. Measurements of ROS, Superoxide, and Lipid Peroxidation

Intracellular ROS level was determined by a DCFH-DA method as previously described [38]. Briefly, the cells were lysed and incubated with DCFH-DA (50 *μ*mol/L) at 37°C for 30 min, and then, the fluorescence intensity was measured at the excitation/emission wavelengths of 485/535 nm. To evaluate the level of superoxide, the cells were incubated with dihydroethidium (10 mmol/L) at 37°C for 30 min, and then, the fluorescence intensity was measured at the excitation/emission wavelengths of 485/530 nm. Malondialdehyde (MDA), an oxidative product of lipid, was measured to evaluate lipid peroxidation using a Lipid Peroxidation Assay Kit. Briefly, the cells were lysed with 303 *μ*L lysis solution on ice and centrifuged to remove insoluble materials, and then, the supernatants were incubated with 600 *μ*L TBA reagent at 95°C for 60 min according to the manufacturer's instructions. Next, the absorbance was measured at 532 nm. Lipid peroxidase (LPO) level was detected using a commercial kit. Briefly, the cells were lysed and reacted with chromogenic reagents at 45°C for 60 min, and then, the absorbance was measured at 586 nm.

### 2.8. Iron Assay

To evaluate intracellular ferrous iron (Fe^2+^) level, the cells were rapidly homogenized in iron assay buffer, centrifuged at 16000 g for 10 min to remove insoluble materials, and then incubated with iron assay buffer at 25°C for 30 min [7, 39]. Next, each well was added with 100 *μ*L iron probe and incubated at 25°C for 60 min. The absorbance was measured at 593 nm. To measure total iron, the samples were mixed with 5 *μ*L iron reducer to reduce Fe^3+^ to Fe^2+^.

### 2.9. Evaluation of Hydroxyeicosatetraenoic Acids (HETEs)

Levels of 5-HETE and 15-HETE in the medium were detected by commercial kits (MyBioSource and Abcam) following the manufacturer's instructions.

### 2.10. Western Blot

Total proteins were extracted, quantified, and denaturalized according to standard protocols [40]. Then, equal amounts of total proteins were separated by 8% SDS-PAGE, transferred to PVDF membranes, and blocked with 5% skim milk. Next, the membranes were incubated with primary antibodies at 4°C overnight and secondary antibodies at room temperature for 1 h [26]. Finally, protein bands were visualized by ECL reagent and quantified by Image Lab software.

### 2.11. Luciferase Reporter Assay

The potential binding site of miR-4735-3p in SCL40A1 3′-UTR was obtained from TargetScan (http://www.targetscan.org/vert_71/), and then, the predicted sequence was cloned into the luciferase reporter vector (Promega, Madison, WI, USA). HEK293T cells were cotransfected with the miR-4735-3p mimic and luciferase reporter plasmid, and relative luciferase reporter activity was determined at 48 h using Dual Luciferase Reporter Assay System (Promega) [41, 42].

### 2.12. Clinical Samples

Clinical ccRCC samples were obtained according to the ethical principles of Declaration of Helsinki as we previously described and then were pathologically confirmed and staged according to WHO classification and 2010 American Joint Committee on Cancer classification [26]. Informed consent was obtained from all the patients. This study was approved by the Ethical Committee of Zhangzhou Affiliated Hospital of Fujian Medical University.

### 2.13. Statistical Analysis

All data were expressed as the mean ± SD and analyzed by SPSS 22.0. Unpaired Student's *t*-test was performed for comparisons between 2 groups, and a one-way ANOVA was used to compare differences among more than 2 groups. *P* < 0.05 was considered statistically significant.

## 3. Results

### 3.1. Expression of miR-4735-3p in Human ccRCC Tissues and Cell Lines

**Figure 1:**
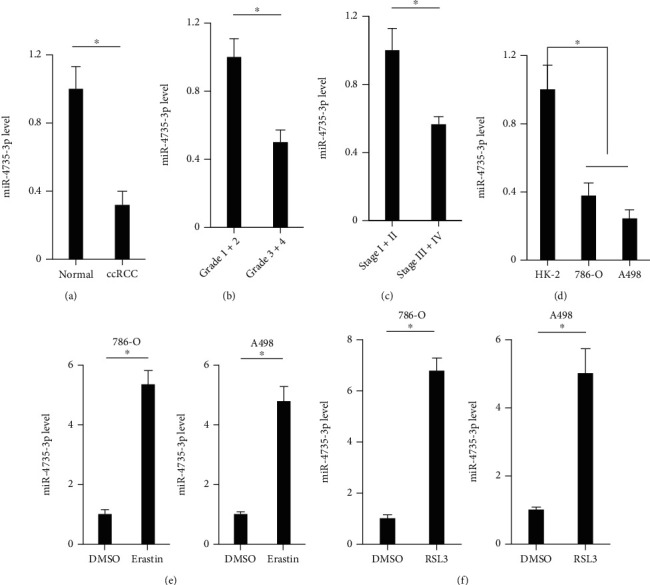
Expression of miR-4735-3p in human ccRCC tissues and cell lines. (a) Relative expression of miR-4735-3p in human ccRCC tissues and adjacent normal tissues. (b) Relative expression of miR-4735-3p in human ccRCC tissues of different histological grades. (c) Relative expression of miR-4735-3p in human ccRCC tissues of different clinical TNM stages. (d) Relative expression of miR-4735-3p in human ccRCC cell lines or normal kidney cell line. (e and f) Relative expression of miR-4735-3p in human ccRCC cell lines upon different ferroptotic stimulations. *N* = 6 per group. All data were expressed as the mean ± SD; ^∗^*P* < 0.05 was considered significant.

To investigate the role of miR-4735-3p in human ccRCC progression, we first detected the expression of miR-4735-3p in human ccRCC tissues and cell lines. As shown in Figure 1(a), miR-4735-3p expression was reduced in human ccRCC tissues. And miR-4735-3p downregulation also correlated with histological grade and clinical TNM stage (Figures 1(a)–1(c)). Besides, miR-4735-3p expression was reduced in human ccRCC cell lines, including 786-O and A498 cells (Figure 1(d)). To validate the potential involvement of miR-4735-3p in ferroptosis of human ccRCC cell lines, 786-O and A498 cells were treated with erastin or RSL3. Intriguingly, miR-4735-3p expression was elevated in human ccRCC cell lines upon ferroptotic stimulation (Figures 1(e) and 1(f)). These findings demonstrate that miR-4735-3p may be involved in the regulation of ferroptosis and tumor progression of human ccRCC.

### 3.2. Overexpression of miR-4735-3p Induces Ferroptosis of Human ccRCC Cell Lines

**Figure 2:**
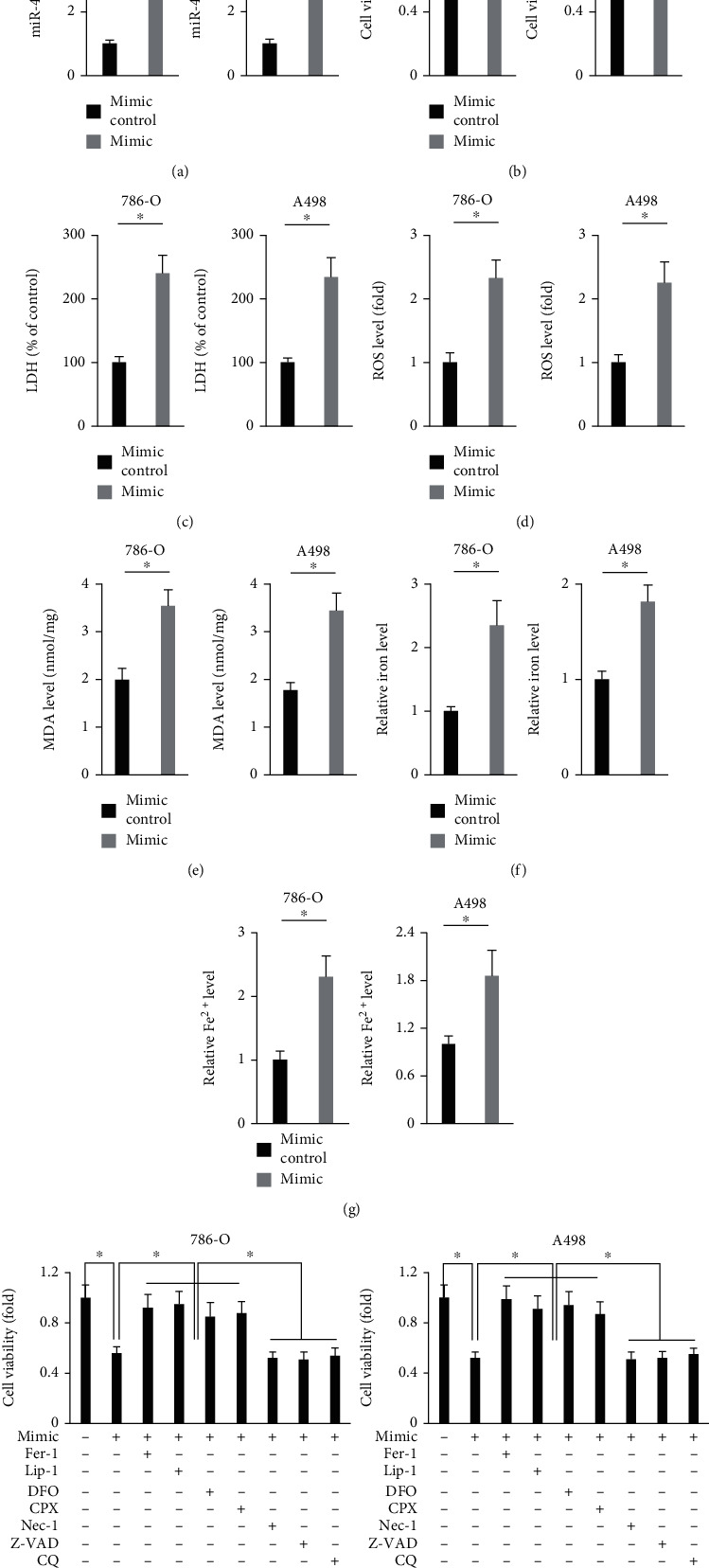
Overexpression of miR-4735-3p induces ferroptosis of human ccRCC cell lines. (a) Relative expression of miR-4735-3p in human ccRCC cell lines treated with the miR-4735-3p mimic or mimic control. (b) Cell viability was examined by the MTT method. (c) Relative LDH level in the medium from the miR-4735-3p mimic or mimic control-treated ccRCC cells. (d and e) Relative ROS and MDA levels. (f and g) Relative intracellular iron and Fe^2+^ levels. (h) Cell viability in the miR-4735-3p mimic-treated ccRCC cells in the presence of various cell death inhibitors. *N* = 6 per group. All data were expressed as the mean ± SD; ^∗^*P* < 0.05 was considered significant.

Then, 786-O and A498 cells were transfected with the miR-4735-3p mimic to overexpress miR-4735-3p in human ccRCC cell lines (Figure 2(a)). As shown in Figure 2(b) and Figure S1A, overexpression of miR-4735-3p significantly reduced survival and colony formation of human ccRCC cell lines. And cell death ratio was also increased by the miR-4735-3p mimic-treated cells, as evidenced by increased LDH releases (Figure 2(c)). In addition, we found that treatment with the miR-4735-3p mimic significantly suppressed the migration and invasion of 786-O and A498 cells (Figure S1B-C). The findings in Figures 1(e) and 1(f) revealed that miR-4735-3p expression in human ccRCC cell lines was elevated by ferroptotic inducers, indicating a potential involvement of miR-4735-3p in ferroptosis; therefore, we investigated whether the miR-4735-3p mimic-induced cell death was ferroptosis. Ferroptosis is a novel form of programmed cell death triggered by an iron-mediated accumulation of toxic lipid ROS [20]. As shown in Figures 2(d) and 2(e) and Figure S1C-D, the levels of intracellular ROS and lipid peroxidation were significantly increased in cells treated with the miR-4735-3p mimic. Excessive ROS causes oxidative damage and fragmentation of polyunsaturated fatty acids (PUFAs) to release HETEs [20, 21]. In accordance with the increased ROS in the miR-4735-3p mimic-treated cells, those cells also displayed higher 5-HETE and 15-HETE levels in the culture medium (Figure S1E-F). Iron overload is a major contributor of ferroptosis, and our data revealed that the miR-4735-3p mimic significantly increased intracellular iron level in human ccRCC cell lines (Figures 2(f) and 2(g)). To further validate the involvement of ferroptosis in the miR-4735-3p mimic-mediated growth inhibition of human ccRCC cell lines, the miR-4735-3p mimic-treated 786-O and A498 cells were incubated with different inhibitors of cell death. As shown in Figure 2(h), it was ferroptotic inhibitors (Fer-1 and Lip-1) and iron chelators (DFO and CPX), instead of necroptotic (Nec-1), apoptotic (Z-VAD) or autophagic (CQ) inhibitors, that significantly prevented the miR-4735-3p mimic-mediated cell death of human ccRCC cell lines. These findings reveal that overexpression of miR-4735-3p induces ferroptosis of human ccRCC cell lines.

### 3.3. Suppression of miR-4735-3p Reduces Ferroptosis of Human ccRCC Cell Lines

**Figure 3:**
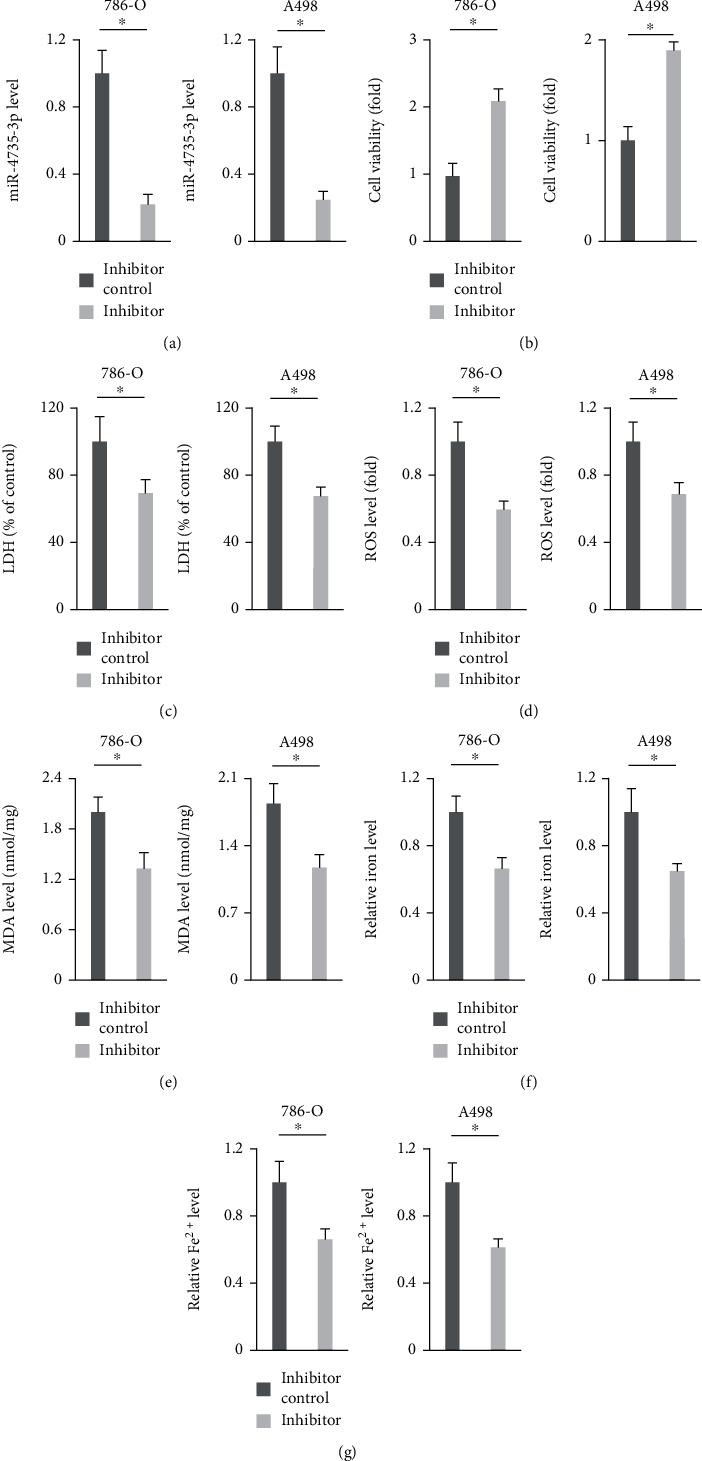
Suppression of miR-4735-3p reduces ferroptosis of human ccRCC cell lines. (a) Relative expression of miR-4735-3p in human ccRCC cell lines treated with the miR-4735-3p inhibitor or inhibitor control. (b) Cell viability was examined by the MTT method. (c) Relative LDH level in the medium from the miR-4735-3p inhibitor or inhibitor control-treated ccRCC cells. (d and e) Relative ROS and MDA levels. (f and g) Relative intracellular iron and Fe^2+^ levels. *N* = 6 per group. All data were expressed as the mean ± SD; ^∗^*P* < 0.05 was considered significant.

Next, we investigated whether suppression of miR-4735-3p could reduce ferroptosis of human ccRCC cell lines using the miR-4735-3p inhibitor (Figure 3(a)). As shown in Figure 3(b) and Figure S2A, viability and colony formation of 786-O and A498 cells were suppressed by the miR-4735-3p inhibitor. And cell death ratio was also reduced in the presence of the miR-4735-3p inhibitor, as evidenced by the decreased LDH releases (Figure 3(c)). In addition, the miR-4735-3p inhibitor also enhanced the migration and invasion of human ccRCC cell lines (Figure S2B-C). As expected, ROS production and lipid peroxidation in human ccRCC cell lines were attenuated by the miR-4735-3p inhibitor (Figures 3(d) and 3(e) and Figure S2D-E). And treatment with the miR-4735-3p inhibitor also blocked oxidative damage of PUFAs, as evidenced by the decreased 5-HETE and 15-HETE levels in the medium (Figure S2F-G). Moreover, iron content was decreased in the miR-4735-3p inhibitor-treated 786-O or A498 cells (Figures 3(f) and 3(g)). These data suggest that suppression of miR-4735-3p reduces ferroptosis of human ccRCC cell lines.

### 3.4. Overexpression of miR-4735-3p Induces Ferroptosis through Downregulating SLC40A1

**Figure 4:**
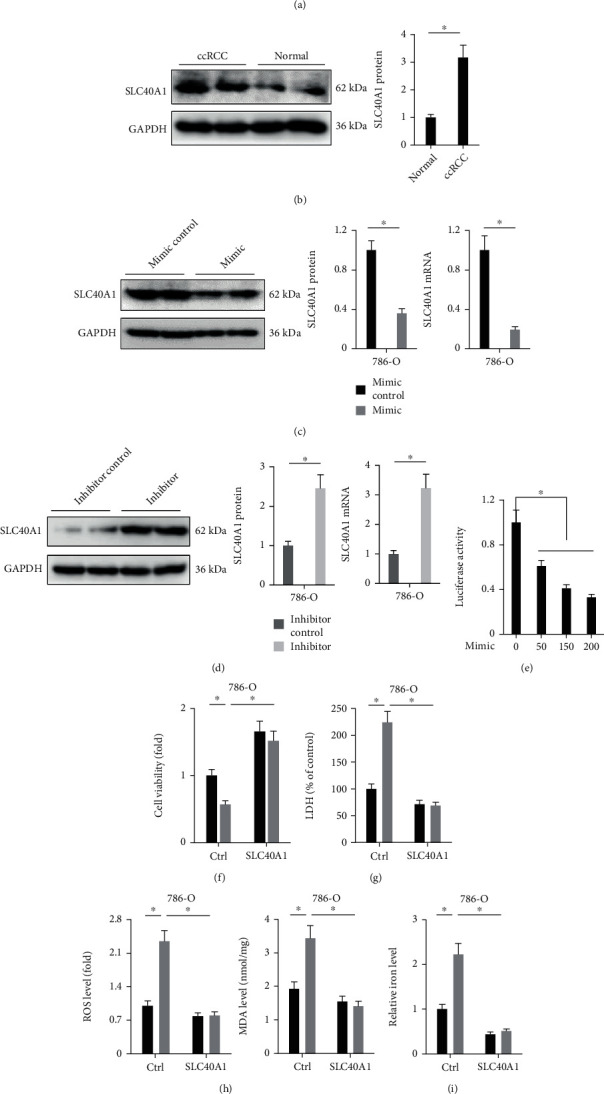
Overexpression of miR-4735-3p induces ferroptosis through downregulating SLC40A1. (a) The complementary sequences of miR-4735-3p in SLC40A1 3′-UTR. (b) Relative SLC40A1 protein level in human ccRCC tissues and adjacent normal tissues. (c and d) Relative SLC40A1 protein and mRNA levels in 786-O cells treated with the miR-4735-3p mimic, inhibitor, or respective controls. (e) Relative luciferase activity in HEK293T cells transfected with the miR-4735-3p mimic and SLC40A1 3′-UTR. (f) Relative viability in SLC40A1-overexpressed 786-O cells treated with the miR-4735-3p mimic or mimic control. (g) Relative LDH releases in 786-O cells. (h) ROS level and MDA content in 786-O cells. (i) Relative iron level in 786-O cells. *N* = 6 per group. All data were expressed as the mean ± SD; ^∗^*P* < 0.05 was considered significant.

Finally, we investigated the specific mechanisms mediating ferroptosis of the miR-4735-3p mimic. Using the TargetScan software, we identified some potential targets of miR-4735-3p, among which SLC40A1 was selected for further study because of its role in regulating intracellular iron homeostasis. As shown in Figure 4(a), a potential complementary sequence of miR-4735-3p was found in SLC40A1 3′-UTR. And the protein level of SLC40A1 was upregulated in human ccRCC tissues, indicating a potential involvement of SLC40A1 in ccRCC progression (Figure 4(b)). In addition, mRNA levels and protein expressions of SLC40A1 in human ccRCC cells were decreased by the miR-4735-3p mimic but increased by the miR-4735-3p inhibitor (Figures 4(c) and 4(d) and Figure S3A). The findings from luciferase reporter assay further validated the direct interaction between miR-4735-3p and SLC40A1 (Figure 4(e)). To clarify the necessity of SLC40A1, 786-O and A498 cells were overexpressed with SLC40A1 before treatment with the miR-4735-3p mimic (Figure S3B). As shown in Figures 4(f) and 4(g) and Figure S3C-D, SLC40A1 overexpression significantly increased cell survival and decreased cell death in the miR-4735-3p mimic-treated 786-O or A498 cells, as evidenced by increased cell viability and decreased LDH releases. In addition, the miR-4735-3p mimic-induced ROS overproduction and lipid peroxidation were also suppressed by SLC40A1 overexpression (Figure 4(h) and Figure S3E). And iron overload in the miR-4735-3p mimic-treated 786-O or A498 cells was also attenuated in those overexpressed with SLC40A1 (Figure 4(i) and Figure S3F-G). In general, we validate that overexpression of miR-4735-3p induces ferroptosis through downregulating SLC40A1.

## 4. Discussion

Incidence of RCC, especially ccRCC, continues to increase recently, accounting for 3%-5% of all adult malignancies, and it is estimated that the newly diagnosed cases of ccRCC is 73750, with the number of deaths reaching 14830 [43]. Despite the improvements of clinical diagnosis and nephrectomy, the outcome of ccRCC patients remains poor. 20%-30% of patients present with metastases at initial diagnosis, and nearly 50% of patients progress to metastasis even undergo surgical treatment. The 5-year survival rate of those patients with metastasis is less than 12% [44]. In the present study, we detected a significant downregulation of miR-4735-3p in human ccRCC tissues and cell lines. Overexpression of miR-4735-3p significantly inhibited, while suppression of miR-4735-3p facilitated growth of human ccRCC cell lines. Intriguingly, we found that the expression of miR-4735-3p in 786-O and A498 cells was upregulated upon ferroptotic stimulation and that treatment with the miR-4735-3p mimic significantly facilitated oxidative stress, lipid peroxidation, and ferroptosis in human ccRCC cell lines. In contrast, the miR-4735-3p inhibitor effectively prevented lipid peroxidation and ferroptosis in 786-O and A498 cells, thereby promoting cell growth. Mechanistic studies revealed that miR-4735-3p directly bound to the 3′-UTR of SLC40A1 and downregulated its expression, resulting in iron overload and ferroptosis. In general, we demonstrate that miR-4735-3p facilitates ferroptosis and tumor suppression in ccRCC by targeting SLC40A1 and that overexpression of miR-4735-3p may prevent human ccRCC progression.

Ferroptosis emerges as a nonapoptotic form of cell death and is involved in the initiation and progression of multiple diseases, including human cancer. Accordingly, pharmacological inducer, especially the endogenous activators, of ferroptosis is a focus of intense investigation in cancer therapy [3, 20]. Ferroptosis is regulated by a series of signaling mechanisms, among which iron homeostasis plays critical roles. It is well accepted that iron enters into cells through the TF/TFR system and is stored in ferritin and then exported to extracellular environment by SLC40A1. Of note, SLC40A1 is the only discovered iron export protein in mammals, and suppressing SLC40A1 leads to iron overload and ferroptosis in various tumor cells [21, 39]. In the present study, we identified a potential complementary sequence of miR-4735-3p in SLC40A1 3′-UTR and determined that the miR-4735-3p mimic significantly reduced SLC40A1 expression, resulting in iron overload, lipid peroxidation, and ferroptosis in human ccRCC cell lines. Actually, targeting endogenous miRs helps to develop a novel field to treat various human cancers, including ccRCC. Our previous studies revealed that miR-423-5p was upregulated in human prostate cancer tissues and cell lines and that miR-423-5p knockdown dramatically inhibited proliferation and tumor growth in vivo and in vitro [45]. In addition, we found that the abundances of miR-99b and miR-99b∗ were lower in human ccRCC tissues and cell lines and that overexpression of miR-99b and miR-99b∗ inhibited the proliferation and migration of ccRCC cells [26]. miR-4735-3p is a well-known tumor-associated miRs and contributes to the progression of various human cancers. Teng et al. and C. Li et al. identified miR-4735-3p as a tumor suppressor in bladder cancer and ovarian cancer and demonstrated that suppression of miR-4735-3p facilitated the progression of these two cancers [28, 30]. Consistently, we herein also showed that overexpression of miR-4735-3p inhibited the growth of human ccRCC cell lines by inducing ferroptosis. Yet, findings from Zhou et al. suggested that overexpression of miR-4735-3p suppressed apoptosis of prostate cancer cells upon docetaxel treatment [27]. And Luo et al. and Wang et al. also defined an oncogenic role of miR-4735-3p in human lung cancer cell lines [29, 46]. These discrepancies of the role of miR-4735-3p in different tumors should be investigated in the future.

In general, we for the first time demonstrate that miR-4735-3p facilitates ferroptosis and tumor suppression in ccRCC by targeting SLC40A1.

## Data Availability

The data that supported the findings of this study are available on reasonable request from the corresponding author.

## Supplementary Materials

Supplementary Materials Figure S1: overexpression of miR-4735-3p induces ferroptosis of human ccRCC cell lines. (A) Colony numbers in the miR-4735-3p mimic or mimic control-treated ccRCC cells. (B and C) Cell migration and invasion as measured by the Transwell assay. (D) Relative superoxide level was determined by dihydroethidium staining. (E) Relative LPO level. (F and G) Quantification of 5-HETE and 15-HETE levels in the medium from the miR-4735-3p mimic or mimic control-treated ccRCC cells. *N* = 6 per group. All data were expressed as the mean ± SD; ^∗^*P* < 0.05 was considered significant. Figure S2: suppression of miR-4735-3p reduces ferroptosis of human ccRCC cell lines. (A) Colony numbers in the miR-4735-3p inhibitor or inhibitor control-treated ccRCC cells. (B and C) Cell migration and invasion as measured by the Transwell assay. (D) Relative superoxide level was determined by dihydroethidium staining. (E) Relative LPO level. (F and G) Quantification of 5-HETE and 15-HETE levels in the medium from the miR-4735-3p inhibitor or inhibitor control-treated ccRCC cells. *N* = 6 per group. All data were expressed as the mean ± SD, ^∗^*P* < 0.05 was considered significant. Figure S3: overexpression of miR-4735-3p induces ferroptosis through downregulating SLC40A1. (A) Relative SLC40A1 mRNA level in in A498 cells treated with the miR-4735-3p mimic, inhibitor, or respective controls. (B) Relative SLC40A1 mRNA level in human ccRCC cell lines infected with adenovirus carrying SLC40A1 or Ctrl. (C) Relative viability in SLC40A1-overexpressed A498 cells treated with the miR-4735-3p mimic or mimic control. (D) Relative LDH releases in A498 cells. (E) ROS level and MDA content in A498 cells. (F) Relative Fe^2+^ level in 786-O cells. (G) Relative iron and Fe^2+^ level in A498 cells. *N* = 6 per group. All data were expressed as the mean ± SD; ^∗^*P* < 0.05 was considered significant.
