# Supplementary material for: MicroRNA-4735-3p Facilitates Ferroptosis in Clear Cell Renal Cell Carcinoma by Targeting SLC40A1
Source: Anal Cell Pathol (Amst). 2022 May 19;2022:4213401. doi: 10.1155/2022/4213401 (PMC9135554; doi:10.1155/2022/4213401)
Supplement: Supplementary Materials — Figure S1: overexpression of miR-4735-3p induces ferroptosis of human ccRCC cell lines. (A) Colony numbers in the miR-4735-3p mimic or mimic control-treated ccRCC cells. (B and C) Cell migration and invasion as measured by the Transwell assay. (D) Relative superoxide level was determined by dihydroethidium staining. (E) Relative LPO level. (F and G) Quantification of 5-HETE and 15-HETE levels in the medium from the miR-4735-3p mimic or mimic control-treated ccRCC cells. N = 6 per group. All data were expressed as the mean ± SD; ∗P < 0.05 was considered significant. Figure S2: suppression of miR-4735-3p reduces ferroptosis of human ccRCC cell lines. (A) Colony numbers in the miR-4735-3p inhibitor or inhibitor control-treated ccRCC cells. (B and C) Cell migration and invasion as measured by the Transwell assay. (D) Relative superoxide level was determined by dihydroethidium staining. (E) Relative LPO level. (F and G) Quantification of 5-HETE and 15-HETE levels in the medium from the miR-4735-3p inhibitor or inhibitor control-treated ccRCC cells. N = 6 per group. All data were expressed as the mean ± SD, ∗P < 0.05 was considered significant. Figure S3: overexpression of miR-4735-3p induces ferroptosis through downregulating SLC40A1. (A) Relative SLC40A1 mRNA level in in A498 cells treated with the miR-4735-3p mimic, inhibitor, or respective controls. (B) Relative SLC40A1 mRNA level in human ccRCC cell lines infected with adenovirus carrying SLC40A1 or Ctrl. (C) Relative viability in SLC40A1-overexpressed A498 cells treated with the miR-4735-3p mimic or mimic control. (D) Relative LDH releases in A498 cells. (E) ROS level and MDA content in A498 cells. (F) Relative Fe2+ level in 786-O cells. (G) Relative iron and Fe2+ level in A498 cells. N = 6 per group. All data were expressed as the mean ± SD; ∗P < 0.05 was considered significant. [file 4213401.f1.docx]

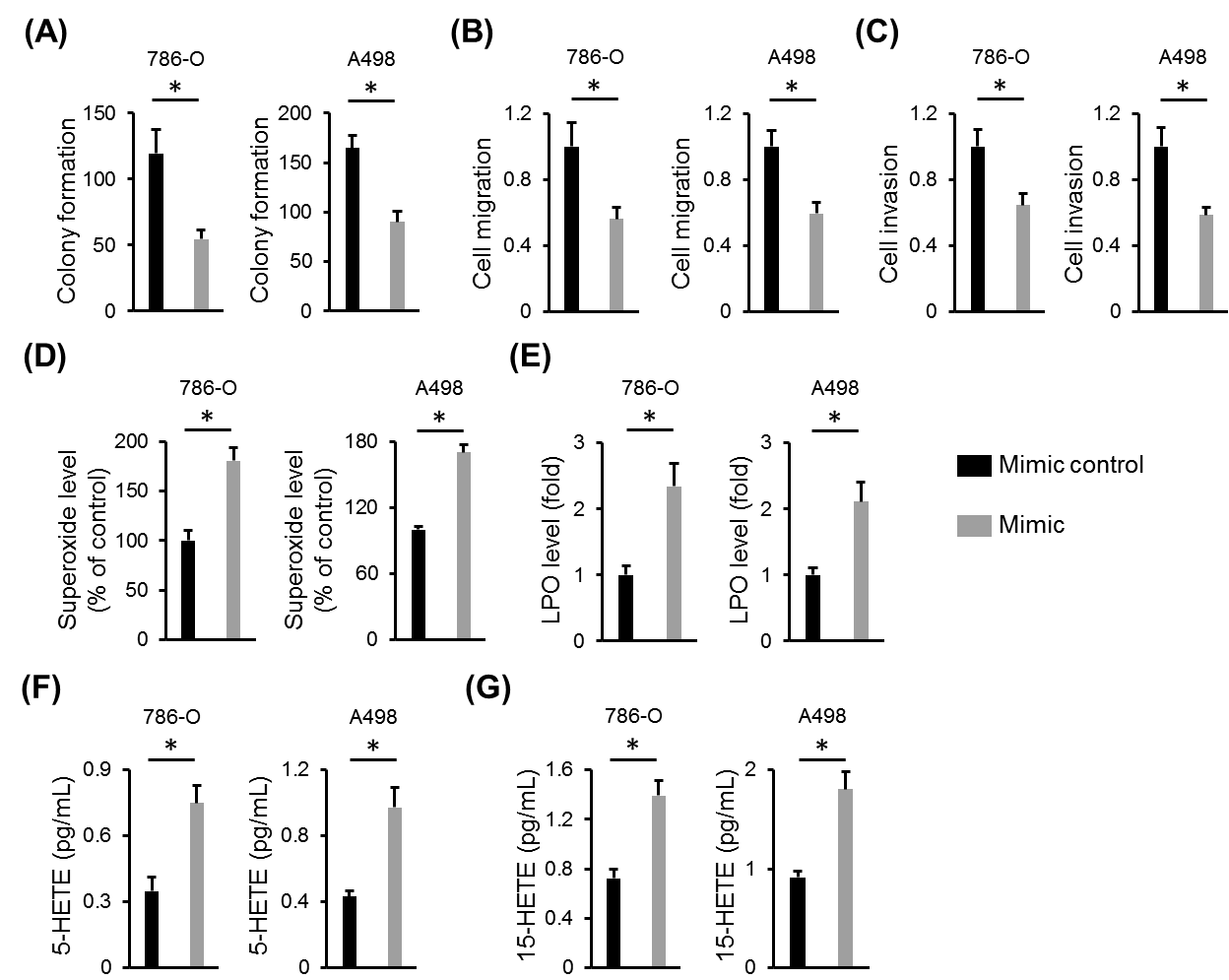


Figure S1. Overexpression of miR-4735-3p induces ferroptosis of human ccRCC cell lines. (A) Colony numbers in the miR-4735-3p mimic or mimic control-treated ccRCC cells. (B-C) Cell migration and invasion as measured by the Transwell assay. (D) Relative superoxide level was determined by dihydroethidium staining. (E) Relative LPO level. (F-G) Quantification of 5-HETE and 15-HETE levels in the medium from the miR-4735-3p mimic or mimic control-treated ccRCC cells. N=6 per group. All data were expressed as the mean ± SD, **P* < 0.05 was considered significant.


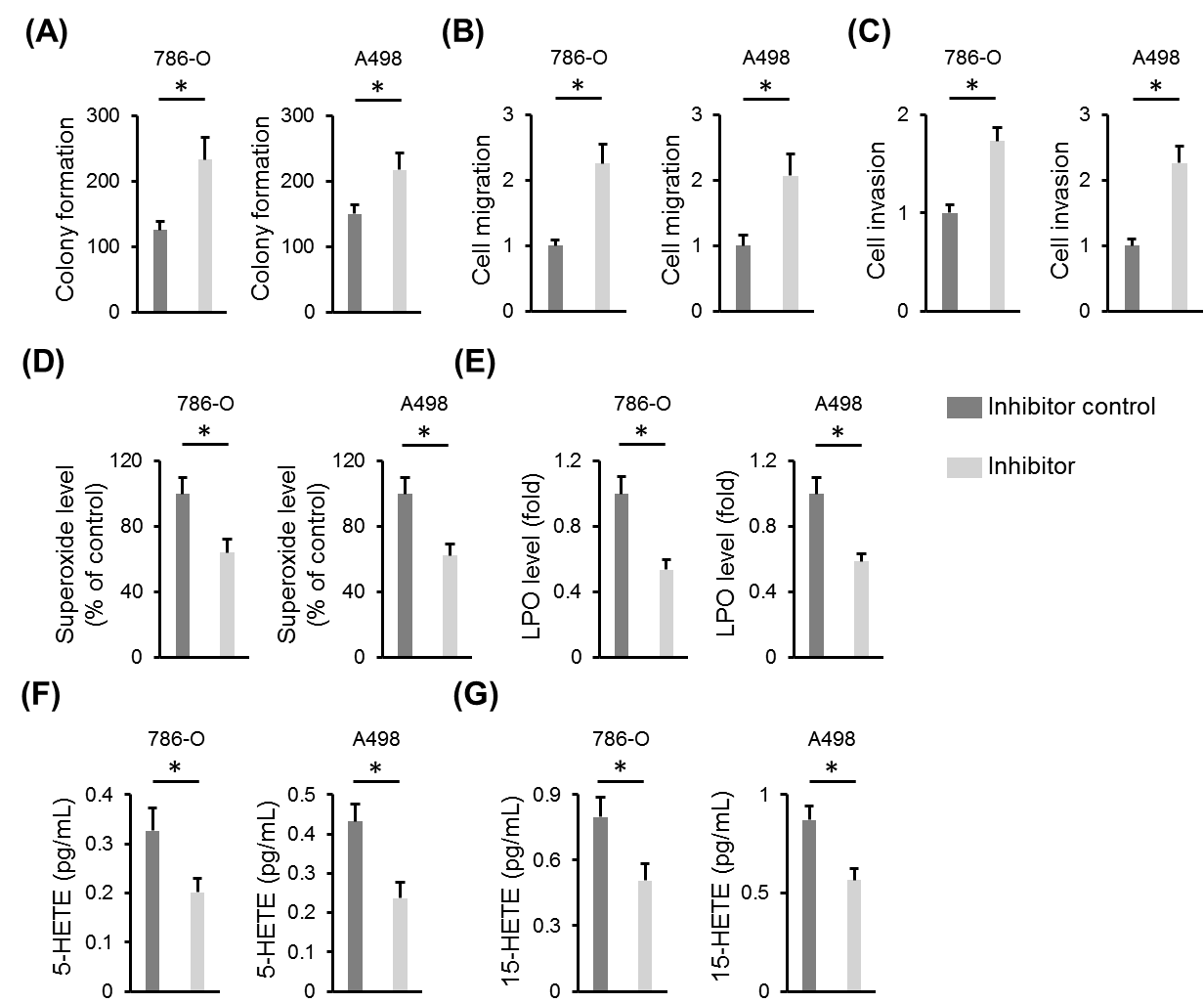


Figure S2. Suppression of miR-4735-3p reduces ferroptosis of human ccRCC cell lines. (A) Colony numbers in the miR-4735-3p inhibitor or inhibitor control-treated ccRCC cells. (B-C) Cell migration and invasion as measured by the Transwell assay. (D) Relative superoxide level was determined by dihydroethidium staining. (E) Relative LPO level. (F-G) Quantification of 5-HETE and 15-HETE levels in the medium from the miR-4735-3p inhibitor or inhibitor control-treated ccRCC cells. N=6 per group. All data were expressed as the mean ± SD, **P* < 0.05 was considered significant.


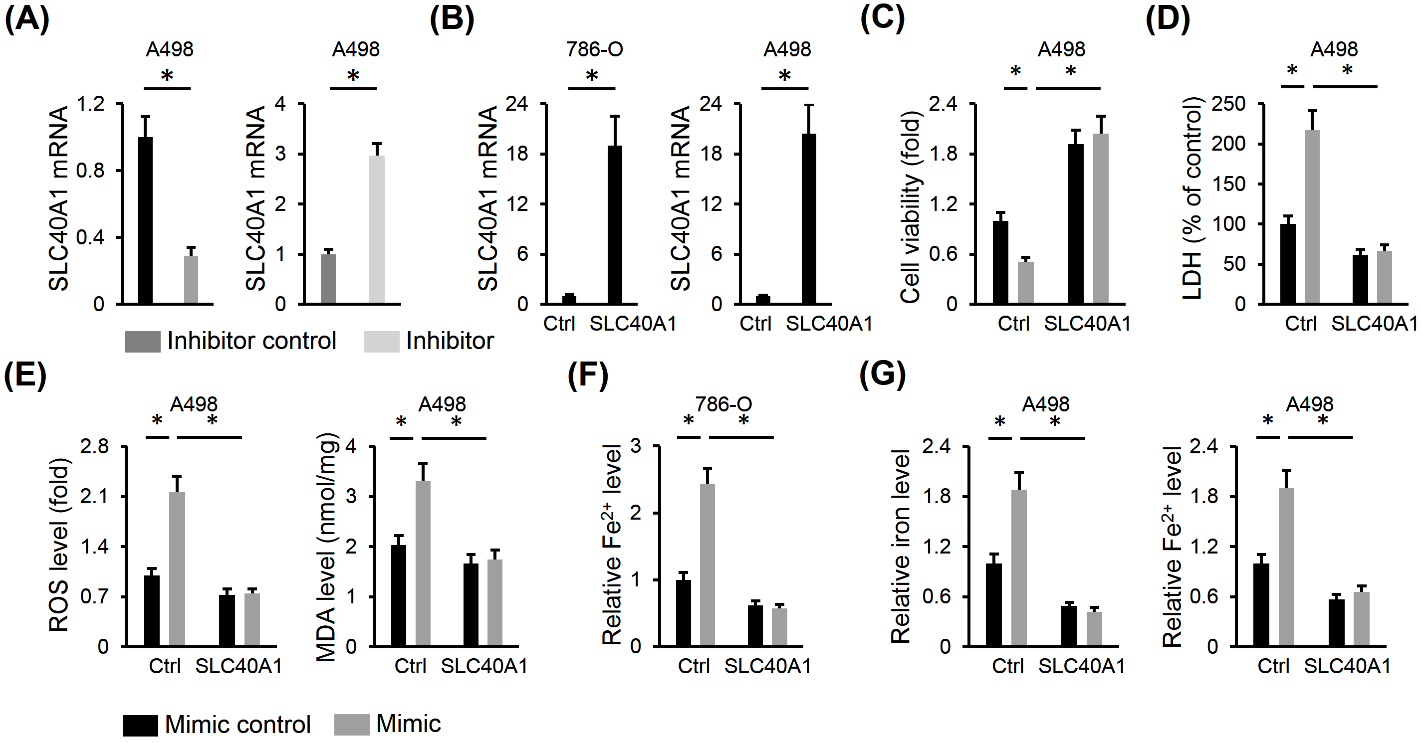


Figure S3. Overexpression of miR-4735-3p induces ferroptosis through downregulating SLC40A1. (A) Relative SLC40A1 mRNA level in in A498 cells treated with the miR-4735-3p mimic, inhibitor or respective controls. (B) Relative SLC40A1 mRNA level in human ccRCC cell lines infected with adenovirus carrying SLC40A1 or Ctrl. (C) Relative viability in SLC40A1-overexpressed A498 cells treated with the miR-4735-3p mimic or mimic control. (D) Relative LDH releases in A498 cells. (E) ROS level and MDA content in A498 cells. (F) Relative Fe^2+^ level in 786-O cells. (G) Relative iron and Fe^2+^ level in A498 cells. N=6 per group. All data were expressed as the mean ± SD, **P* < 0.05 was considered significant.
